# A theory-based analysis of the implementation of online asynchronous telemedicine platforms into primary care practices using Normalisation Process Theory

**DOI:** 10.1186/s12875-025-02717-0

**Published:** 2025-02-06

**Authors:** Cara Leighton, Natalie Joseph-Williams, Annavittoria Porter, Adrian Edwards, Alison Cooper

**Affiliations:** 1 Cardiff University School of Medicine, Cardiff, UK; 2 Division of Population, Medicine Cardiff University, Associate Director Health and Care Research Wales Evidence Centre, 8th Floor, Neuadd Meirionnydd, Heath Park, Cardiff, CF14 4YS UK; 3 Division of Population, Medicine Cardiff University, PRIME Centre Wales and Health and Care Research Wales Evidence Centre, 8th Floor, Neuadd Meirionnydd, Heath Park, Cardiff, CF14 4YS UK

**Keywords:** Primary care, Family medicine, General practice, Asynchronous telemedicine, Implementation, Theory-based analysis, Normalisation Process Theory

## Abstract

**Background:**

Online asynchronous telemedicine platforms are effective and have been implemented in primary care practices, but it is unclear whether implementation was successful. Implementation has not been studied on a large scale in primary care practice. Normalisation Process Theory is a sociological theory used to understand how complex practices can be embedded into routine practice. We aimed to identify and evaluate factors affecting, and make recommendations for, implementation of online asynchronous telemedicine platforms in primary care practice using Normalisation Process Theory.

**Methods:**

A systematic search was carried out across four databases. Studies included were empirical research, published between January 2015 and November 2022, of qualitative, quantitative and mixed methods designs, focusing on implementation of online asynchronous telemedicine platforms designed for two-way secure communication between patients and healthcare professionals to give or receive medical advice in primary care. Data extraction was guided by the domains of Normalisation Process Theory: context, mechanisms, outcomes.

**Results:**

25 reports from 21 primary studies were obtained. COVID-19 changed the context in which asynchronous platforms were implemented into primary care, due to restrictions on face-to-face contact. Coherence is supported by online platforms providing benefits for patients. Healthcare staff felt confident using platforms and better teamworking added to cognitive participation, however patient ‘misuse’ of platforms hindered this. Collective action was negatively affected by poor usability and integration of platforms into practice systems. Reflexive action through large- and small-scale studies had allowed improvements to be made, but poor response rates inhibit this. Outcomes include changed roles and responsibilities for staff and patients and high patient satisfaction. There are concerns regarding confidentiality and health inequities.

**Conclusions:**

Increased workload, lack of integration into existing systems and poor usability affect implementation. Widespread implementation of online platforms in primary care practices can be supported by policy-makers through consistent guidelines to improve platforms’ content, functionality and compatibility with clinical systems to try to enable improvements in practice. Further research should explore patient groups or needs for which online platforms are most suitable, reasons why online platforms work better for different patients and how different patient groups can be supported to benefit from asynchronous telemedicine.

**Supplementary Information:**

The online version contains supplementary material available at 10.1186/s12875-025-02717-0.

**Table:** 

Text box 1. Contributions to the literature
• Research shows online asynchronous telemedicine platforms can be effective and comparable to face-to-face and telephone consultations [1].
• Patients and healthcare professionals agree asynchronous platforms have the potential to be beneficial and reduce pressure on primary care services but problems with implementation have emerged including poor integration into existing systems and lack of usability are barriers to sustained implementation.
• We made recommendations for policy-makers to improve implementation of platforms through better integration into clinical systems, ensure up to date guidelines and prioritise user feedback in platform development, and recommendations to improve implementation in practice ensuring better workflow and accessibility of platforms.
• We identified that further research should address differences in asynchronous platform use between patient groups.

## Background

**Fig. 1:**
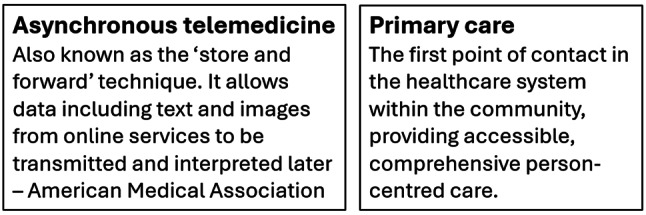
Definitions: asynchronous telemedicine and primary care. Asynchronous telemedicine definition adapted from the American Medical Association definition [2]. Primary care definition adapted from NHS England and the World Health Organisation [3, 4].

Online asynchronous telemedicine platforms (defined in Fig. 1) can be comparable to face-to-face and telephone consultations when used in primary care practices, (defined in Fig. 1) and are effective in providing timely care, prescribing medications, and resolving patients’ problems [1]. The COVID-19 pandemic led to a rapid increase in asynchronous telemedicine uptake in primary care, by patients and healthcare professionals but it is unclear whether this was effectively implemented and whether this led to continued use [1]. 

Several studies report issues with efficiency of asynchronous telemedicine platforms in primary care, including increased staff workload and barriers to workflow, including lack of integration into existing primary care systems, lack of communication between staff and poor usability of online systems, which suggests online platforms may have been implemented poorly [1, 5]. There are suggestions asynchronous telemedicine can lead to increased patient demand, also contributing to increased workload [1]. However, other studies report opposite effects of asynchronous telemedicine on workflow, and promotion of teamworking, suggesting there is sometimes better implementation of these systems [6]. 

This study will use results from a systematic literature search to evaluate factors affecting implementation of online asynchronous platforms in primary care, which may help explain these differing reports in the literature. Previously, implementation of asynchronous telemedicine in primary care has only been evaluated on a small scale [7, 8], whereas synchronous telemedicine has been widely studied in a primary care setting, with a recent systematic review looking at feasibility of implementation [9]. 

In other areas of healthcare asynchronous telemedicine has been successfully implemented and widely used, particularly in dermatology [10], resulting in high levels of diagnostic accuracy, comparable to face-to-face consultations [11]. A theory-based approach to evaluating teledermatology implementation, including a systematic review, led to improved understanding of its implementation, and has potentially helped enable wider use of these systems [12, 13]. A theoretical approach provides a framework for evaluating factors that affect implementation. Normalisation Process Theory (NPT) will be used as it assists in understanding the contextual influences, the mechanisms and outcomes of implementation and so can inform the wider implementation of asynchronous telemedicine platforms in primary care, and future research in this area [14]. 

### What is Normalisation Process Theory?

Normalisation Process Theory is a sociological theoretical model used to understand how complex practices are implemented, embedded, and integrated to become normal or routine [18]. This model was originally designed to explain the implementation of complex interventions in healthcare and its development involved studies of implementing multiple interventions, including teledermatology, which shows it is applicable to asynchronous telemedicine. It is now widely accepted and used for studying healthcare technologies and was chosen for this study.

The context, mechanisms and outcomes affect the implementation process of introducing an intervention into routine practice [14]. The implementation context is the social structure into which an intervention is being integrated. Implementation mechanisms can be broken down into four constructs: coherence, cognitive participation, collective action, and reflexive monitoring, as modelled by May et al., all of which are required for an intervention to be embedded into routine practice [14, 15]. Coherence requires individuals involved having a shared understanding and motivation to adopt the intervention. Cognitive participation is the drive to implementation and is reliant on individuals’ commitment to their roles. Collective action concerns how well individuals involved in implementation work together, whether that is healthcare staff, patients or the intervention itself. Individual or group assessments of the intervention, whether formal or informal, are known as reflexive monitoring. Implementation outcomes are practical changes made to implementation and are influenced by the implementation mechanisms.

### Aims

The primary aim of this study was to identify and evaluate factors affecting implementation of online asynchronous telemedicine platforms in primary care practices as barriers or facilitators to implementation using the framework of NPT.

The secondary aim was to make recommendations for the implementation of online asynchronous telemedicine platforms into general practice.

## Methods

### Design

This study is a systematic review, with theory-based analysis of quantitative and qualitative research studies from primary care settings.

### Search strategy

The search was carried out across four databases: Medline, CINAHL, Embase and Scopus, after completing a pilot Medline search. Search terms were based on three themes: primary care practice, asynchronous telemedicine, and care outcomes. Appendix 1 details full search strategies. Searches were completed in November 2022. Empirical research studies published between January 2015 and November 2022 were included, to cover literature published before and following the COVID-19 pandemic outbreak in March 2020, as this is when a dramatic increase in the implementation and use of online platforms occurred. Citation searching of included studies was carried out to identify further relevant studies.

### Eligibility criteria

The eligibility criteria, outlined in Table 1, were developed using population, intervention, comparison and outcomes (PICO) framework [16], and incorporated the domains of healthcare quality [17]. The focus of this review was on the factors affecting implementation of online asynchronous telemedicine platforms used for secure two-way communication between patients and healthcare professionals in primary care practices.

**Table 1:** Inclusion and exclusion criteria

	Inclusion	Exclusion
Population	Patients and staff who have used asynchronous telemedicine in a primary care/general practice/family medicine setting. Including all healthcare professionals and other members of staff and consultations relating to all patient groups, including adults, children, and carers.	Dentistry, optometry, community nursing, pharmacy. Secondary and tertiary care.
Intervention	Online platforms designed for two-way secure communication between patients and healthcare professionals for the purpose of giving or receiving medical advice. E.g., e-consults, secure messaging, eVisits. Interactions between patients and healthcare professionals seeking medical advice.	Synchronous telemedicine such as video appointments, telephone appointments. Text and email consultations. Automated asynchronous telemedicine, telemonitoring, interactions between two or more healthcare professionals.
Comparison	Face to face consultations Synchronous telemedicine No comparison Before, and following the outbreak of the COVID-19 pandemic	
Outcomes	Uses of asynchronous telemedicine. Safety • Adverse events, harm caused or medical errors. Timeliness • Time to appointment. Effectiveness • Diagnosis made or resolution of problem. • Treatments delivered e.g., prescribed medication. • Number of appointments arranged and attended following asynchronous consultation and the type of follow up. Efficiency • Effect on workflow for healthcare professionals and patients, cost effectiveness • Reduction or replacement of other types of consultations. Equitability • Access for patients. Patient-centeredness • Perceptions of patients and healthcare professionals.	
Study Design	Empirical research: Quantitative studies – comparative and observational studies Mixed methods studies Qualitative studies	Healthcare policies Editorials and opinion pieces Case studies Study protocols
Other	English language Studies including data from 2015 onwards. Studies involving healthcare systems that are comparable to the NHS, for example, OECD countries.	

### Study selection

Screening of search results against inclusion criteria was carried out by one researcher (CL) and 10% was independently screened to check agreement (AP), with queries or disagreements discussed within the research team. Full texts were screened by one researcher (CL). Search results and inclusion decisions were examined by co-authors and recorded using EndNote 20 [18]. 

### Data extraction and analysis

The three domains of Normalisation Process Theory affect implementation: contexts, mechanisms and outcomes. Data were extracted to map to these domains. The four constructs of implementation mechanisms (coherence, cognitive participation, collective action and reflexive monitoring) were used as a model to develop understanding of the implementation of online asynchronous telemedicine platform in primary care practices, with consideration of each of the 16 sub-constructs of the theory [14, 15, 19]. Data were extracted from the included studies by one researcher (CL) and examined by co-authors.

## Results

### Search results

**Fig. 2:**
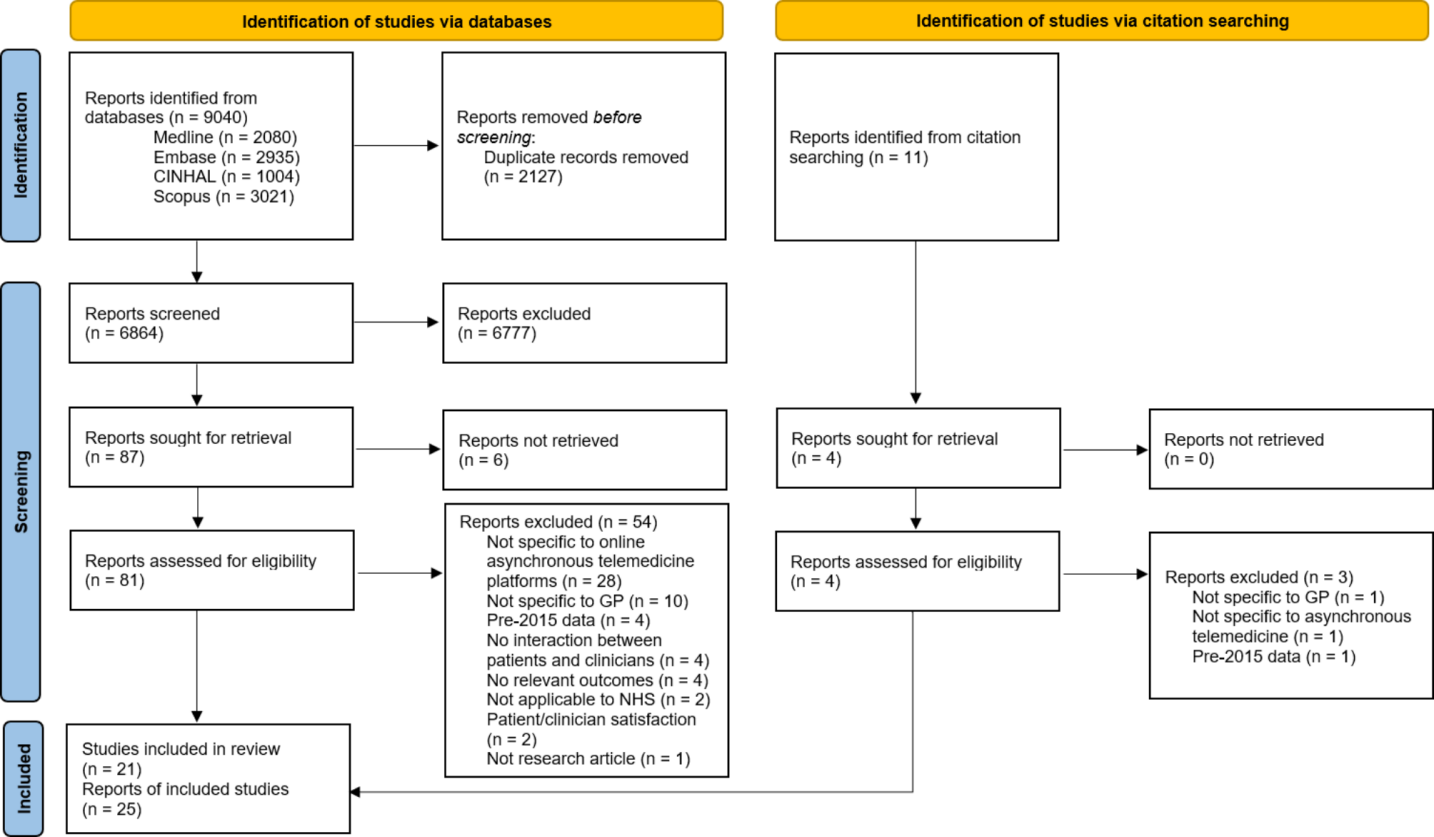
PRISMA flow diagram. Flow diagram outlining the systematic search and screening process. Adapted from PRISMA [20].

9040 reports were retrieved through database searching. There were 6864 reports after removing duplicates, of which 6777 were excluded through title and abstract screening. Of the remaining 87, 81 were retrieved and assessed for eligibility and six were inaccessible. 11 further records were retrieved through citation searching; following abstract and title screening four were assessed for inclusion. Overall, 25 reports from 21 primary studies (*n* = 21) were included. A preferred reporting items for systematic reviews and meta-analyses (PRISMA) diagram (Fig. 2) summarises the screening process [20]. Details of the reasons for exclusion of full texts are recorded in Appendix 2.

### Study characteristics

Included studies (*n* = 21) were from a range of countries; United Kingdom (*n* = 9), United States (*n* = 5), Spain (*n* = 2), Sweden (*n* = 1), The Netherlands (*n* = 1), Norway (*n* = 1), Canada (*n* = 1), Israel (*n* = 1). Eight studies involved interviews of patients and/or staff, six involved a cross-sectional survey, and some involved free text responses for qualitative analysis, two involved other cross-sectional data, two involved collection of other qualitative data, ten were retrospective cohort studies involving quantitative data, with one also involving prospective data. Five included studies reported on implementation during the COVID-19 pandemic, with four comparing from before to during the COVID-19 pandemic.

### Types of online platforms

Online platforms are used in different ways within the included studies. All included studies involved online platforms that could be used for two-way communication for medical advice and administrative requests initiated by patients. Some involved patient questionnaires [6, 8, 21–28], some used decision support algorithms, for example recommending an antibiotic to prescribe [6, 25, 26], and some were part of a wider platform including services like video-consulting [29, 30]. One platform was for patients with a limited number of conditions [31], and another had options for healthcare professionals or patients to initiate consultations, which led to it being used for mass messaging to promote the flu vaccine campaign during the COVID-19 pandemic [32–34]. Some platforms included the option to upload pictures, videos and audio files [8, 23, 24, 27]. Some platforms required patients to register before using it [28, 29], and one practice advertised the introduction of online platforms via a poster in the practice [22]. 

### Implementation context

*The implementation context concerns the social structures that an intervention is being implemented within*,* in this case the intervention is online asynchronous telemedicine platforms*,* and the social structure is primary care practices*,* which encompasses the relationships between staff members and how practice policies and procedures and use of online systems affect these relationships.*

For successful implementation to occur online platforms must be integrated into primary care practices’ existing IT and clinical systems and their use must be incorporated into staff workflow, with clear roles for healthcare professionals and administration staff. Patients must also understand how to access and use the platform and both healthcare staff and patients should have access to suitable internet connection [35]. 

The implementation context differed before and during the COVID-19 pandemic. Telemedicine, including use of online asynchronous platforms was implemented rapidly and on a large scale due to limitations on face-to-face contact, with some governments, for example the UK government, instructing primary care practitioners to move almost exclusively remote consulting [7, 36, 37]. This led to easier access in the UK as many companies offered online asynchronous platforms at low or zero cost [7]. In Spain, an online platform that previously required patients to be authorised to use it became available to all patients and additional permissions were added, for example allowing remote approval of sick notes, also leading to easier access [34]. 

Following the resolution of the COVID-19 pandemic it is not clear how the context of implementation of online asynchronous platforms in primary care has changed compared to before and during, owing to lack of direct comparisons, but differing context could explain why some practices did not plan to continue using asynchronous consultations following the pandemic [37]. 

### Implementation mechanisms

1. **Coherence** – *relates to how well patients*,* healthcare professionals and administrative staff understand their role in using the intervention and how well they understand its value and benefits.*

The perceived benefit of asynchronous telemedicine for staff is reducing or replacing telephone and face-to-face consultations, making general practice more efficient [38]. One study reported developers and healthcare professionals viewed that healthcare should follow other industries, like banking, and move online, as technology will solve existing problems [39]. There are suggestions this works best for chronic conditions [5]. 

While differences are expected when multiple platforms are being developed in different healthcare systems, confusion could occur if multiple platforms are implemented within the same service, hindering coherence [8, 23, 24]. There was a disparity in staff roles found across the included studies, which could also hinder coherence. In some practices clinical staff carried out triaging of e-consultations; deciding whether clinical review, further consultation, or administrative tasks were required [5, 8, 22–24], but in others administrative staff carried this out [39]. 

A lack of clarity around patients’ roles was found, with differences in how staff and patients thought online platforms should be used [21]. One study reported patients submitted requests for asynchronous consultations that GPs thought clearly needed a face-to-face appointment and GPs felt there was a lower threshold for patients to seek appointments using online consultations, increasing workload [8, 22–24]. This links to the view some staff had, that asynchronous consultations are inefficient [35]. Another study had low uptake of asynchronous consultations from patients, and some staff put this down to existing systems working for patients, also hindering coherence [22]. 

2. **Cognitive participation*** concerns how implementation of a new intervention is driven and how individuals’ commitment (patients and healthcare staff) to carrying out their roles adds to this.*

Healthcare professionals’ confidence in responding to online consultations affects their participation. Some found it difficult not having verbal cues [5]. One study found GPs with more experience in telephone triage were more confident with asynchronous consultations and therefore arranged fewer face-to face follow up consultations [8]. 

Practices with better teamworking were better able to implement online platforms [5], and staff felt a sense of pride in working together to implement platforms [7]. For some platforms healthcare staff and patients were involved in the design and implementation [8, 39], with administrative staff particularly showing investment in implementing online platforms by taking on triaging roles in some practices that support clinicians in the responsibility for clinical decision making [39]. 

Healthcare professionals reported patients ‘misusing’ online systems, for example, to get faster access to face-to-face appointments or by repeatedly sending e-consultations [5, 8, 23, 24, 39]. One study found patients used the system to make complaints [39]. There were also problems around patients not following advice given following e-consultation, including booking recommended follow up appointments, deterring ongoing implementation [30]. 

3. **Collective action*** involves how well patients*,* healthcare staff and the online platform itself work together within primary care practices.*

GPs thought online platforms were good for straightforward queries and for mental health problems and found they had a better starting point for follow-up consultations, as they had the patient’s presenting complaint and medical history from the e-consultation and existing record [8, 23, 24]. 

Two studies found patients thought online consultations were easier than phoning to book appointments [8, 23, 24]. One study found platforms that were more easily accessible on GP websites had higher usage of online consultations [27]. However, lack of usability of online platforms for patients hinders implementation. For one platform, many patients stated they had to downplay symptoms to prevent the system displaying a ‘phone 999’ (emergency call) message [27], and others complained platforms involving questionnaires were laborious, repetitive, and sometimes their problems did not fit [6, 27]. 

Some patients found they had to repeat information due to being followed up by multiple GPs for the same request, but this was not a problem for one practice which chose to route e-consultations to the most suitable healthcare professional [5], and another where the platform included an option to choose a preferred GP [8, 23, 24]. 

Another hindering factor is when online platforms are not integrated well into existing practice systems. One study found a lack of information flow from online platforms to primary care clinical records [5], and another found implementation was hindered when platforms recommended antibiotic prescriptions that were not in line with local or national guidelines [25, 26]. 

4. **Reflexive monitoring** – *concerns the ongoing appraisal and evaluation of an intervention.*

One platform included an optional patient feedback survey which consisted of free text and tick box questions involving Likert-like scales [8, 27]. Other studies involved stand-alone appraisals of online platforms, including large qualitative and quantitative studies [5, 8, 23–26, 29, 30, 32–35, 37, 38, 40, 41], and smaller scale evaluations, for example in individual practices [6, 7, 21, 22, 27, 28, 31, 39]. 

In total 14 included studies involved feedback from healthcare staff and patients through qualitative interviews and questionnaires, allowing improvements to be made. For example, one practice adjusted the time given for e-consultations, from three per ten-minute consultation slot to one [39], and one platform had features added, including the option to consult for multiple symptoms in one consultation, upload photos, choose a preferred GP, addition of a separate channel for admin requests and language was simplified [8, 23, 24, 27]. 

Factors inhibiting appraisal of online asynchronous telemedicine, and therefore working against implementation, include poor response rates to staff and patient questionnaires, and lack of availability for interviews [5, 27, 30, 35, 37]. One problem with surveying patients who have completed online consultations is that this does not reach patients who do not use the platforms, so important learning points may be missed.

### Implementation outcomes

*Implementation outcomes are practical changes made to implementation as a result of the implementation mechanisms.*

Some studies reported the introduction of online platforms led to administrative staff carrying out a triaging role, changing their responsibilities [39], but potentially reducing pressure on clinical staff [8]. However, this increased their workload, with staff developing a shared understanding not to disturb each other when carrying out e-consultation work [39]. Clinical staff roles also diversified with one study reporting GPs could be more involved in managerial and commercial ventures [39]. 

There is also a suggestion that online platforms put more responsibility on patients, with some feeling more engaged and empowered by this method of consulting [5]. Some platforms required patients to register to use it, and while many did register this did not always lead to them using the online consultation services [28, 29]. 

Many benefits for patients were found including flexibility, convenience and improved access as asynchronous platforms are available 24 h a day, saving travel time and costs and reducing embarrassment or worries about discussing certain medical problems [5, 8, 21, 23, 24]. Waiting times were also reduced as responses must be received within one or two working days [8, 23, 24]. This is significant for patients in full time employment or with childcare responsibilities who may find it difficult to attend face-to-face consultations [6, 27, 35], and is enhanced by the option to upload pictures, videos and audio files [8, 23, 24, 27]. These findings will further improve coherence as staff and patients understand the benefits that asynchronous platforms provide.

Patients were satisfied with their experiences using online platforms and the response times, but they preferred general questionnaires to symptom- or condition-specific ones [39]. Staff were less satisfied as they had not experienced the reduction in workflow expected from introducing online platforms, but recognised the juxtaposition of implementation problems and the benefits they provide patients [22]. Poor integration of platforms into IT systems and staff workflow led to staff having to find a work-around to stop patients receiving appointment confirmation texts when adding an e-consultation to the GP’s list in one study [39], and another found staff spent time making phone calls to patients who were not aware they should expect one [21]. Healthcare staff were also concerned that pre-existing health inequities causing some patients to be digitally excluded, could potentially be worsened by implementing online platforms [6, 7]. One study found staff thought patient involvement would aid implementation [27]. These factors affecting staff may have contributed to one study finding practices did not intend to continue using asynchronous platforms [37]. 

Both patients and healthcare professionals had concerns about privacy and confidentiality [35]. One patient was surprised to receive a response from a receptionist as they thought their information would only be viewed by their GP [22], and other patients were unhappy their request was not seen by a doctor [8, 23, 24].

#### Recommendations for policy, practice, and research

Table 2 outlines our recommendations for policy, practice and future research and illustrating how they tie in with the theoretical model of NPT.

**Table 2:** Recommendations for policy, practice and future research

	Recommendation	NPT construct
Policy	Online asynchronous platforms should be compatible with existing general practice IT systems and patient records.	Implementation context
	To improve platform usability patients and healthcare staff who are users of the platform should be involved in the development and implementation of platforms.	Implementation mechanisms – coherence
	In the UK introducing only one online platform across all GP practices would reduce confusion and improve implementation and collaboration between practices.	Implementation mechanisms – coherence
	Platforms should have the option to send messages to all patients to improve mass messaging for campaigns, such as for flu vaccines.	Implementation mechanisms – coherence
	There should be guidance detailing which members of staff carry out which roles and training should be provided to ensure staff confidence.	Implementation mechanisms – cognitive participation
	Platforms involving algorithms or suggested outcomes to guide healthcare staff should be updates regularly, in line with national and local guidelines.	Implementation mechanisms – reflexive monitoring
Practice	Individual practices should identify members of staff to be responsible for certain roles to ensure streamlined workflow.	Implementation mechanisms – coherence
	Platforms should be easily accessible for patients via practice websites.	Implementation mechanisms – coherence
	Platforms should be available to patients 24 h a day.	Implementation mechanisms – coherence
	Practices and platforms should make it clear to patients what type of queries they should use asynchronous platforms for and what responses they can expect, including whether to expect a phone call or check for an online response.	Implementation mechanisms - coherence
Future research	There should be further research into reasons that patient register for online platforms but do not go on to use the services.	Implementation mechanisms –reflexive monitoring
	There should be further research into factors that might be stopping patients from accessing and using asynchronous telemedicine and how to overcome these.	Implementation mechanisms –reflexive monitoring
	Research should identify what type of patients are using asynchronous telemedicine and when it works for them, whether this is specific patient groups of condition specific.	Implementation outcomes

Our recommendations focus on three key areas that are important for the development, future implementation and sustainability of online asynchronous telemedicine platforms: healthcare policy, practice and future research.

Recommendations for policy focus on the actions that can be undertaken by healthcare policy makers, for example, those in government, to improve implementation of online platforms and make their use more sustainable. Our recommendations include improving integration into existing primary care IT and clinical systems, ensuring that platforms are in line with healthcare guidelines and prioritising feedback from those who use the platforms in their ongoing development.

Recommendations for practice focus more on the actions that individual practices, or individual staff can undertake to improve implementation, including improving workflow, and ensuring platforms are accessible and usable for their patients.

Our recommendations for future research focus on patient use of online platforms, including which patient groups are using asynchronous platforms, or for which needs, and the barriers and facilitators to this.

## Discussion

### Principal findings

Online asynchronous telemedicine platforms have been implemented into primary care practices in different ways, with COVID-19 changing this context dramatically, making telemedicine a necessity due to restrictions on face-to-face contact.

The benefits of implementing online platforms for patients are well recognised, including improved flexibility, convenience and access, but the perceived benefits for staff were not always seen in practice. A lack of clarity around administrative staff and patients’ roles in using online platforms hinders coherence. Healthcare staff confidence in using online platforms affected cognitive participation, and it was found practices with better teamworking were better able to implement these platforms. However, patient ‘misuse’ of platforms negatively affected this. Poor integration of platforms into practice systems and platforms being hard for patients to navigate hindered implementation, indicating contextual constraints which led to poor collective action. However, there were examples of platforms without these issues, indicating implementation can be enhanced by platform design. Reflexive monitoring is occurring in the implementation of online platforms as they have been appraised on both large and small scales, including qualitative and quantitative studies involving patients and healthcare staff, allowing improvements to be made. Factors inhibiting reflexive monitoring include poor response rates in qualitative studies.

Outcomes of implementing asynchronous online platforms into primary care practices include changed roles and responsibilities for healthcare staff and patients and staff having to find work-arounds to compensate for poor integration in practice systems. Appraisals of online platforms have found patients are mainly satisfied with their experiences. However, staff concerns include the potential for reinforcing existing health inequities for digitally excluded patients and both staff and patients worried about privacy and confidentiality.

### Context of existing literature

Factors promoting and inhibiting implementation of synchronous telemedicine and teledermatology are consistent with our findings, including improved access and efficiency, lack of compatibility with existing systems, technological limitations, and communication difficulties. There was also evidence of organisational resistance to change, which suggests this hinders implementation of all types of telemedicine in primary care practices [9, 12, 42]. 

There was evidence that inclusion of patients’ and healthcare professionals’ feedback assisted in implementation of teledermatology, so it is important that this is continued in the evaluation and implementation of asynchronous telemedicine platforms [12]. 

Convenience of services for patients was shown to be a promoting factor in implementing synchronous telemedicine and teledermatology, which is consistent with our findings [9, 12, 13]. However, studies have not addressed other patient factors that may be barriers or facilitators to implementation, including socioeconomic status, preferred language, age and technical literacy, which have been shown to influence patient uptake of synchronous telemedicine [9, 42]. This would be important to study; particularly as asynchronous consulting is rapidly evolving and therefore the ways platforms are used and the patient groups targeted may change.

Finally, the results of this review and theory-based analysis add to the results of our previous review, which reported issues with workflow and workload affected the efficiency and therefore effectiveness of asynchronous telemedicine use in primary care [1]. Our results show that in some cases improvements have been made to address these issues and our recommendations outline further ways this can be improved upon in the ongoing implementation of asynchronous online platforms in primary care settings. A gap in the literature we identified in our previous review was a lack of economic analysis of online asynchronous platforms, which is also an important factor to consider within the context of implementation [1]. 

### Strengths and limitations

A strength of this study is it used evidence from a wide range of online platforms in general practice settings in different countries, which were identified through a systematic search of the literature.

Using NPT is a strength of this review as it is a widely accepted and used model for studying the implementation of complex healthcare technologies. However, it is important to recognise this could have restricted our results as there could be other factors affecting implementation that do not fit into this model.

A limitation is that data extraction and analysis were carried out by only one researcher, but this was minimised by examining findings and discussing the interpretation of results in the NPT framework within the research team, who were experienced in using this model.

### Policy, practice, and research

The recommendations outlined in this study can be used to influence the design and development of new and existing online asynchronous platforms and develop guidelines for the use of these systems. They also outline improvements that can be made in practice to aid implementation in practice of online platforms for healthcare professionals and patients. Finally, they identify gaps in the existing literature that would benefit from further research. Further research should particularly study differences between socio-economic and condition-specific groups of patients for their reasons for using or not using asynchronous platforms. Asynchronous consulting is rapidly evolving and therefore the ways and patient groups that could use asynchronous platforms most effectively may change.

## Conclusion

The main challenges affecting implementation of online asynchronous platforms in primary care practices are increased workload for staff, lack of integration into existing clinical systems, and lack of usability for patients. Patients are satisfied with the convenience that online platforms provide and there are success stories including the rapid implementation that occurred following the outbreak of the COVID-19 pandemic. For online asynchronous telemedicine platforms to be implemented more widely into primary care practices there needs to be action from policy-makers to improve platforms and provide consistent guidance and training for those involved in its use. There needs to be further research into factors affecting patients’ uptake of telemedicine, for which patient groups it works and how to overcome difficulties that some patients face.

## Electronic supplementary material

Below is the link to the electronic supplementary material.

Supplementary Material 1

Supplementary Material 2

Supplementary Material 3

## Data Availability

The full search strategy used for each database is available in Appendix 1 of the additional materials. The data extracted from included studies according to the 12 propositions of NPT are available from the corresponding author on reasonable request.

## Abbreviations

CINAHL: Cumulated Index to Nursing and Allied Health Literature – a literature database
GP: A term used in the UK for general practice or general practitioner, similar to a family doctor or primary care doctor in international healthcare systems
IT: Information technology
NHS: National Health Service – the public healthcare service in the UK
NPT: Normalisation Process Theory
PICO: Population, intervention, comparison, outcomes – framework used for inclusion and exclusion criteria in systematic reviews
PRISMA: Preferred Reporting Items for Systematic Reviews and Meta-Analyses
UK: United Kingdom
US: United States
