# Supplementary material for: A theory-based analysis of the implementation of online asynchronous telemedicine platforms into primary care practices using Normalisation Process Theory
Source: BMC Prim Care. 2025 Feb 6;26:27. doi: 10.1186/s12875-025-02717-0 (PMC11800456; doi:10.1186/s12875-025-02717-0)
Supplement: Supplementary file 1 — Supplementary Material 1 [file 12875_2025_2717_MOESM1_ESM.pdf]

Study title:

A theory-based analysis of the implementation of online asynchronous telemedicine platforms into primary care practices using Normalisation Process Theory

Corresponding author:

Cara Leighton, BSc - Medical Student, Cardiff University School of Medicine, leightonch@cardiff.ac.uk
ORCID: 0009-0002-3881-0298

Appendix 1 – Strategies from systematic search

Medline

1. Exp Telemedicine/
2. Exp remote Consultation/
3. (telemedic* or tele-medic*).tw.
4. (e-visit or evisit or electronic visit).tw.
5. (asynchronous adj3 (telehealth* or tele-health* or ehealth or e-health or electronic health)).tw.
6. (remote adj2 (consult* or visit)).tw.
7. Secure messag*.tw.
8. (e-consult* or electronic consult* or econsult*).tw.
9. (text adj2 (consult* or appoint* or messg*)).tw.
10. (email adj2 (consult* or appoint*)).tw.
11. 1 or 2 or 3 or 4 or 5 or 6 or 7 or 8 or 9 or 10
12. Exp Geneal Practice/
13. Exp Family Practice/
14. Primary care.tw.
15. Primary health*.tw.
16. General practi*.tw.
17. Family practi*.tw.
18. Family doctor.tw.
19. 12 or 13 or 14 or 15 or 16 or 17 or 18
20. Exp Treatment Outcome/
21. Exp “Quality of Health Care”/
22. (quality or effective*).tw.
23. Exp “Cost and Cost Analysis”/
24. Exp Time-to-treatment/
25. Exp Waiting Lists/
26. Cost*.tw.
27. Exp Patient Safety/
28. Exp Medical Errors/
29. Exp Patient Harm/
30. Error*.tw.
31. (adverse adj2 (incident or event)).tw.
32. 20 or 21 or 22 or 23 or 24 or 25 or 26 or 27 or 28 or 29 or 30 or 31
33. 11 and 19 and 32
34. Limit 33 to English language
35. Limit 34 to yr=”2015 -Current”

Embase

1. Exp Telemedicine/
2. Exp teleconsultation/
3. (telemedic* or tele-medic*).tw.
4. (e-visit or evisit or electronic visit).tw
5. (asynchronous adj3 (telehealth* or tele-health* or ehealth or e-health or electronic health)).tw.
6. (remote adj2 (consult* or visit)).tw.
7. Secure messag*.tw.
8. (e-consult* or electronic consult* or econsult*).tw
9. (text adj2 (consult* or appoint* or messag*)).tw.
10. (email adj2 (consult* or appoint*)).tw.
11. 1 or 2 or 3 or 4 or 5 or 6 or 7 or 8 or 9 or 10
12. Exp general practice/
13. Primary care.tw.
14. Primary health*.tw.
15. General practi*.tw.
16. Family practi*.tw.
17. Family doctor.tw.
18. 12 or 13 or 14 or 15 or 16 or 17
19. Exp treatment outcome/
20. Exp health care quality/
21. (quality or effective*).tw.
22. Exp “coat benefit analysis”/
23. Exp “cost effectiveness analysis”
24. Exp time to treatment/
25. Cost*.tw.
26. Exp patient safety/
27. Exp medical error/
28. Exp patient harm/
29. (adverse adj2 (incident or event)).tw.
30. Error*.tw.
31. 19 or 20 or 21 or 22 or 23 or 24 or 25 or 26 or 27 or 28 or 29 or 30
32. 11 and 18 and 31
33. Limit 32 to (English language and yr=”2015 -Current”)

CINHAL

1. (MH “Telemedicine+”)
2. (MH “Remote Consultation”)
3. TI telemedic* OR AB telemedic* OR TI tele-medic OR AB tele-medic*
4. TI e-visit OR AB e-visit OR TI evisit OR AB evisit OR TI electronic visit OR AB electronic visit
5. TI asynchronous OR AB asynchronous
6. TI asynchronous N3 telehealth* OR AB asynchronous N3 telehealth OR TI asynchronous N3 e-health OR AB asynchronous N3 e-health
7. TI remote N2 consult* OR AB remote N2 consult* OR TI remote N2 visit OR AB remote N2 visit
8. TI secure messag* OR AB secure messag*
9. TI e-consult* OR AB e-consult* OR TI electronic consult* OR AB electronic consult*

10. TI text N2 consult* OR AB text N2 consult OR TI text N2 appoint* OR AB text N2 appoint* OR TI text N2 messag* OR AB text N2 messag*
11. TI email N2 consult* OR AB email N2 consult* OR TI email N2 appoint* OR AB email N2 appoint*
12. 1 OR 2 OR 3 OR 4 OR 5 OR 6 OR 7 OR 8 OR 9 OR 10 OR 11
13. (MH “Family Practice”)
14. TI primary care OR AB primary care
15. TI primary health* OR AB primary health*
16. TI general practi* OR AB general practi*
17. TI family practi* OR AB family practi*
18. TI family doctor OR AB family doctor
19. 13 OR 14 OR 15 OR 16 OR 17 OR 18
20. (MH "Treatment Outcomes+") OR (MH "Outcomes (Health Care)+")
21. (MH “Quality of Health Care+”)
22. TI quality OR AB quality OR TI effective* OR AB effective*
23. (MH "Costs and Cost Analysis+") OR (MH "Cost Benefit Analysis")
24. (MH “Waiting Lists”)
25. TI cost* OR AB cost*
26. (MH “Patient Safety+”)
27. (MH “Health Care Errors+”)
28. (MH “Treatment Errors+”)
29. TI patient harm OR AB patient harm
30. TI adverse N2 event OR AB adverse N2 event OR TI adverse N2 incident OR AB adverse N2 incident
31. TI error* OR AB error*
32. 20 OR 21 OR 22 ORT 23 OR 24 OR 25 OR 26 OR 27 OR 28 OR 29 OR 30 OR 31
33. 12 AND 19 AND 32
34. Limiters – Published date:20150101-20221131; English Language

<u>Scopus</u>

(TITLE-ABS-KEY (general AND practi*) AND PUBYEAR > 2014)

OR (TITLE-ABS-KEY (family AND practi*) AND PUBYEAR > 2014)

OR (TITLE-ABS-KEY (family AND doctor) AND PUBYEAR > 2014)

AND

((TITLE-ABS-KEY (cost*) OR (TITLE-ABS-KEY (cost* W/2 analysis) AND PUBYEAR > 2014))

OR (TITLE-ABS-KEY (time AND to AND treatment) AND PUBYEAR > 2014)

OR (TITLE-ABS-KEY (waiting W/2 list) AND PUBYEAR > 2014)

OR (TITLE-ABS-KEY (quality W/2 health*) AND PUBYEAR > 2014)

OR (TITLE-ABS-KEY (treatment AND outcome*) AND PUBYEAR > 2014)

OR (TITLE-ABS-KEY (medical AND error*) AND PUBYEAR > 2014)

OR (TITLE-ABS-KEY (patient AND safety) AND PUBYEAR > 2014)

OR ((TITLE-ABS-KEY adverse W/2 event*) OR (TITLE-ABS-KEY (adverse W/2 incident*)) AND PUBYEAR > 2014)

OR ((TITLE-ABS-KEY (quality) OR TITLE-ABS-KEY (effective*)) AND PUBYEAR > 2014))

AND

((TITLE-ABS-KEY (email* W/2 consult*) OR TITLE-ABS-KEY (email* W/2 appoint*)) AND PUBYEAR > 2014)

OR ((TITLE-ABS-KEY (text W/2 consult*) OR TITLE-ABS-KEY (text W/2 appoint*) OR TITLE- ABS-KEY (text W/2 messag*) AND PUBYEAR > 2014)

OR ((TITLE-ABS-KEY (e-consult*) OR TITLE-ABS-KEY (econsult*) OR TITLE-ABS-KEY (electronic AND consult*)) AND PUBYEAR > 2014)

OR (TITLE-ABS-KEY (secure AND messag*) AND PUBYEAR > 2014)

OR ((TITLE-ABS-KEY (remote W/2 consult*) OR TITLE-ABS-KEY (remote W/2 visit)) AND PUBYEAR > 2014)

OR ((TITLE-ABS-KEY (asynchron* W/3 ehealth) OR TITLE-ABS-KEY (asynchron* W/3 e-health) OR TITLE-ABS-KEY (asynchron* W/3 electronic AND health) OR TITLE-ABS-KEY (asynchron* W/3 tele-health*) OR TITLE-ABS-KEY (asynchron* W/3 telehealth*)) AND PUBYEAR > 2014)

OR ((TITLE-ABS-KEY (e-visit) OR TITLE-ABS-KEY (evisit) OR TITLE-ABS-KEY (electronic AND visit)) AND PUBYEAR > 2014)

OR ((TITLE-ABS-KEY (Telemedic*) ORT ITLE-ABS-KEY (tele-medic*)) AND PUBYEAR > 2014 AND PUBYEAR > 2014))
