# Supplementary material for: A theory-based analysis of the implementation of online asynchronous telemedicine platforms into primary care practices using Normalisation Process Theory
Source: BMC Prim Care. 2025 Feb 6;26:27. doi: 10.1186/s12875-025-02717-0 (PMC11800456; doi:10.1186/s12875-025-02717-0)
Supplement: Supplementary file 2 — Supplementary Material 2 [file 12875_2025_2717_MOESM2_ESM.pdf]

Study title:

A theory-based analysis of the implementation of online asynchronous telemedicine platforms into primary care practices using Normalisation Process Theory

Corresponding author:

Cara Leighton, BSc - Medical Student, Cardiff University School of Medicine, leightonch@cardiff.ac.uk
ORCID: 0009-0002-3881-0298

Appendix 2 – Excluded studies and reasons for exclusion

Author and year	Journal	DOI	Reason for exclusion
Atherton et al. 2018	British Journal of General Practice	10.3399/bjgp18X694853	Not specific to online asynchronous telemedicine platforms
Baron-Miras et al. 2022	International Journal of Environmental Research and Public Health	10.3390/ijerph192114119	Not specific to online asynchronous telemedicine platforms
Baughman et al. 2022	Obesity Science and Practice	10.1002/osp4.625	Not specific to online asynchronous telemedicine platforms
Benson et al. 2021	Postgraduate Medicine	10.1080/00325481.2021.1934492	Not specific to online asynchronous telemedicine platforms
Bliss et al. 2022	Journal of primary care and community health	10.1177/21501319221114831	Not specific to online asynchronous telemedicine platforms
Bruns et al. 2022	Journal of pharmacy practice	10.1177/08971900221136629	Not specific to online asynchronous telemedicine platforms
Carrier et al. 2022	International Journal of Environmental Research and Public Health	10.3390/ijerph19106220	Not specific to online asynchronous telemedicine platforms
Chavez et al. 2022	Journal of the American Board of Family Medicine	10.3122/jabfm.2022.03.210520	Not specific to online asynchronous telemedicine platforms
Chen et al. 2022	PLoS ONE	10.1371/journal.pone.0272605	Not specific to online asynchronous telemedicine platforms
Coffman et al. 2016	Journal of the American Board of Family Medicine	10.3122/jabfm.2016.04.150375	Not specific to online asynchronous telemedicine platforms
Darley et al. 2022	medRxiv	10.1101/2022.02.21.22271185	Not specific to online asynchronous telemedicine platforms
Dash et al. 2016	BMC Health Services Research	10.1186/s12913-016-1776-9	Not specific to online asynchronous telemedicine platforms
Dhaliwal et al. 2022	Journal of the American Association of	10.1097/JXX.0000000000000626	Not specific to online asynchronous telemedicine platforms

	Nurse Practitioners		
Ekman et al. 2019	International Journal of Medical Informatics	10.1016/j.ijmedinf.2019.04.016	Not specific to online asynchronous telemedicine platforms
Falgarone et al. 2022	Journal of Medical Internet Research	10.2196/33507	Not specific to online asynchronous telemedicine platforms
Gomez et al. 2021	Journal of the American Board of Family Medicine	10.3122/jabfm.2021.S1.200517	Not specific to online asynchronous telemedicine platforms
Hardie et al. 2022	British Journal of General Practice Open	10.3399/BJGPO.2021.0123	Not specific to online asynchronous telemedicine platforms
Hoskin et al. 2021	Future Healthcare Journal	10.7861/fhj.2021-0023	Not specific to online asynchronous telemedicine platforms
Jonnagaddala et al. 2021	International Journal of Medical Informatics	10.1016/j.ijmedinf.2021.104470	Not specific to online asynchronous telemedicine platforms
Kludacz-Alessandri et al. 2021	Journal of Clinical Medicine	10.3390/jcm10163502	Not specific to online asynchronous telemedicine platforms
McGrail et al. 2017	Journal of Medical Internet Research	10.2196/jmir.7374	Not specific to online asynchronous telemedicine platforms
Mold et al. 2015	British Journal of General Practice	10.3399/bjgp15X683941	Not specific to online asynchronous telemedicine platforms
Mold et al. 2019	JMIR Medical Informatics	10.2196/13042	Not specific to online asynchronous telemedicine platforms
Neves et al. 2021	Journal of Telemedicine and Telecare	10.1177/1357633X211066235	Not specific to online asynchronous telemedicine platforms
Olayiwola et al. 2020	JMIR Public Health and Surveillance	10.2196/19045	Not specific to online asynchronous telemedicine platforms
Parker et al. 2021	British Journal of General Practice Open	10.3399/BJGPO.2021.0040	Not specific to online asynchronous telemedicine platforms
Quinlan et al. 2018	Irish Medical Journal		Not specific to online asynchronous telemedicine platforms
Rosen et al. 2022	British Journal of General Practice Open	10.3399/BJGPO.2021.0204	Not specific to online asynchronous telemedicine platforms
Alexander et al. 2020	JAMA Network Open	10.1001/jamanetworkopen.2020.21476	Not specific to general practice
Chan et al. 2018	Journal of general internal medicine	10.1007/s11606-018-4637-x	Not specific to general practice
Erdmann et al. 2022	JAMA Network Open	10.1001/jamanetworkopen.2022.0348	Not specific to general practice
Jetty et al. 2021	Journal of the American Board of Family Medicine	10.3122/jabfm.2021.S1.200202	Not specific to general practice
Nguyen et al. 2021	Journal of Medical Internet Research	10.2196/27531	Not specific to general practice
Rastogi et al. 2020	Journal of general internal medicine	10.1007/s11606-019-05415-7	Not specific to general practice

Shimada et al. 2017	Journal of the American Medical Informatics Association	10.1093/jamia/ocx021	Not specific to general practice
Uscher-Pines et al. 2020	Journal of Medical Internet Research	10.2196/22727	Not specific to general practice
Waschkau et al. 2022	International Journal of Environmental Research and Public Health	10.3390/ijerph192214860	Not specific to general practice
Yakushi et al. 2020	Permanente Journal	10.7812/TPP/19.177	Not specific to general practice
Bavafa et al. 2018	Management Science	10.1287/mnsc.2017.2900	Pre-2015 data
Bertelsen et al. 2015	Studies in health technology and informatics	10.3233/978-1-61499-564-7-376	Pre-2015 data
Huygens et al. 2018	British Medical Journal Open	10.1136/bmjopen-2017-019233	Pre-2015 data
Riippa et al. 2015	Journal of Medical Internet Research	10.2196/jmir.4487	Pre-2015 data
Baldwin et al. 2017	Journal of the American Board of Family Medicine	10.3122/jabfm.2017.05.170088	No interaction between clinician and patient
Balestrieri et al. 2020	Family Medicine	10.1093/fampra/cmaa077	No interaction between clinician and patient
Eccles et al. 2019	British Journal of General Practice	10.3399/bjgp19X702197	No interaction between clinician and patient
Mehta et al. 2019	Journal of general internal medicine	10.1007/s11606-019-05241-x	No interaction between clinician and patient
Anderson et al. 2017	The Annals of Family Medicine	10.1370/afm.2036	Patient/clinician satisfaction
Fagerlund et al. 2019	British Medical Journal Open	10.1136/bmjopen-2018-028251	Patient/clinician satisfaction
Baines et al. 2020	Journal of Medical Internet Research	10.2196/19375	No relevant outcomes
Segui et al. 2021	Journal of Medical Internet Research	10.2196/29622	No relevant outcomes
Zhong et al. 2018	Health Care Management Science	10.1007/s10729-017-9404-8	No relevant outcomes
De Moraes et al. 2022	BMC Research Notes	10.1186/s13104-022-06197-8	Not applicable to NHS
Peabody et al. 2019	Journal of the American Board of Family Medicine	10.3122/jabfm.2019.06.190081	Not applicable to NHS
Join et al. 2020	Journal of the American Medical Association Network Open	10.1001/jamanetworkopen.2020.28392	Not a research article
Roig Cabo et al. 2021	Atencion Primaria	10.1016/j.aprim.2021.102070	Not in English
Barton et al. 2018	Telemedicine and e-Health	10.1089/tmj.2017.0184	Not retrieved
Kim et al. 2015	American Journal of Managed Care		Not retrieved
Motz et al. 2020	South Dakota Medicine: The Journal of the South Dakota		Not retrieved

	State Medical Association		
Murray et al. 2020	Telemedicine and e-Health	10.1089/tmj.2019.0044	Not retrieved
Penza et al. 2021	Telemedicine and e-Health	10.1089/tmj.2020.0047	Not retrieved
Terekhova et al. 2017	British Colombia Medical Journal		Not retrieved
Peeters et al. 2016*	Journal of Medical Internet Research	10.2196/medinform.4515	Pre-2015 data
Player et al. 2018*	Health Affairs	10.1377/hlthaff.2018.05122	Not specific to general practice
Schers et al. 2021*	Annals of Family Medicine	10.1370/afm.2625	Not specific to asynchronous telemedicine

*Retrieved through citation searching
